# Patterns of follow‐up care in adult blood cancer survivors—Prospective evaluation of health‐related outcomes, resource use, and quality of life

**DOI:** 10.1002/cam4.7095

**Published:** 2024-03-29

**Authors:** Hildegard Lax, Julia Baum, Nils Lehmann, Anja Merkel‐Jens, Dietrich W. Beelen, Karl‐Heinz Jöckel, Ulrich Dührsen

**Affiliations:** ^1^ Institut für Medizinische Informatik, Biometrie und Epidemiologie Universität Duisburg‐Essen Essen Germany; ^2^ Klinik für Hämatologie Universitätsklinikum Essen, Universität Duisburg‐Essen Essen Germany; ^3^ Klinik für Knochenmarktransplantation Universitätsklinikum Essen, Universität Duisburg‐Essen Essen Germany

**Keywords:** follow‐up study, health‐related quality of life, hematologic neoplasm, survivorship

## Abstract

**Background:**

Information about follow‐up care in blood cancer survivors is limited. The questionnaire‐based “Aftercare in Blood Cancer Survivors” (ABC) study aimed to identify patterns of follow‐up care in Germany and compare different types of follow‐up institutions.

**Methods:**

The study's 18‐month prospective part compared the follow‐up institutions identified in the preceding retrospective part (academic oncologists, community oncologists, primary care physicians). The questionnaires were completed by the follow‐up physicians.

**Results:**

Of 1070 physicians named by 1479 blood‐cancer survivors, 478 (44.7%) consented to participate. For provision of care, most oncologists relied on published guidelines, while most primary care physicians depended on information from other physicians. Survivors with a history of allogeneic transplantation or indolent lymphoma were mainly seen by academic oncologists, whereas survivors with monoclonal gammopathy, multiple myeloma, or myeloproliferative disorders were often seen by community oncologists, and survivors with a history of aggressive lymphoma or acute leukemia by primary care physicians. Detection of relapse and secondary diseases was consistently viewed as the most important follow‐up goal. Follow‐up visits were most extensively documented by academic oncologists (574 of 1045 survivors cared for, 54.9%), followed by community oncologists (90/231, 39.0%) and primary care physicians (51/203, 25.1%). Relapse and secondary disease detection rates and the patients' quality of life were similar at the three institutions. Laboratory tests were most often ordered by academic oncologists, and imaging by primary care physicians. Psychosocial issues and preventive care were more often addressed by primary care physicians than by oncologists.

**Conclusions:**

Patients at high risk of relapse or late complications were preferentially treated by academic oncologists, while patients in stable condition requiring continuous monitoring were also seen by community oncologists, and patients with curable diseases in long‐term remission by primary care physicians. For the latter, transfer of follow‐up care from oncologists to well‐informed primary care providers appears feasible.

## 1 INTRODUCTION

Due to improvements in diagnosis and treatment, the number of long‐term cancer survivors is continuously growing.^1^, ^2^ Because of the risk of relapse and late effects, follow‐up care is an important component of long‐term support.^3^ How best to provide this support, is a matter of debate.^4^

There are different models of follow‐up care delivery.^5^, ^6^, ^7^ Follow‐up can be provided by follow‐up clinics, medical specialists, general practitioners, and specialized nurses, alone or in combination.^4^ The most common combined model is the parallel type where follow‐up care is provided by oncologists and other health care by general practitioners.^6^, ^7^ In the sequential model, often realized in pediatric oncology, cancer survivors are formally transferred from oncologists to primary care providers.^6^, ^7^ Another, more complex delivery type is the shared‐care model where oncologists and general practitioners have complementary roles in follow‐up care.^5^, ^6^, ^7^

For blood cancer, the fourth most common cancer,^8^, ^9^ little is known about follow‐up care.^10^ Blood cancer differs from solid tumors by its disseminated nature, an unusually large number of subtypes,^11^, ^12^ and a leading role of drug‐ and cell‐based therapies.^13^, ^14^ Surgery, the mainstay of treatment for curable solid tumor stages, has no role in the therapeutic armamentarium of blood cancer.^15^ Whether early recognition of relapse is associated with improved prognosis, remains uncertain for most subtypes.^16^ Given the favorable prognosis of many types of blood cancer, there is a wealth of information about long‐term treatment side effects,^16^, ^17^ secondary diseases,^18^, ^19^, ^20^, ^21^ and quality of life.^22^, ^23^, ^24^, ^25^ How and by whom follow‐up care is delivered, however, remains largely unexplored. Expert opinion‐based recommendations from national and international guidelines remain vague in this respect.^26^, ^27^, ^28^, ^29^ Studies comparing different follow‐up models have not yet been performed for blood cancer,^3^ and comprehensive information about blood cancer follow‐up practice patterns in Germany is not available.

To gather information about the follow‐up care received by blood cancer survivors from the University Hospital of Essen, the oldest and one of the largest comprehensive cancer centers in Germany, and to compare patterns of care, we performed a questionnaire‐based observational study consisting of two parts. The retrospective part of the “Aftercare in Blood Cancer Survivors” (ABC) study aimed at identifying follow‐up institutions. It was based on information provided by 1551 blood cancer survivors with a median follow‐up time of almost 10 years. The most important result of this part of the study was the identification of three distinct groups of care providers: academic oncologists working at the university hospital, community oncologists working outside the university hospital, and non‐oncological internists or general practitioners.^30^ In the German health care system, academic and community oncologists receive the same training. Depending on the time period when the qualification was obtained, oncologists in training spend 6 (before 2006) or 5 years (after 2006) in various fields of internal medicine with an additional 2 years in hematology and medical oncology. Major differences between academic and community oncologists include specific diagnostic and therapeutic procedures and access to specialized inpatient care and the expertise of other disciplines that are more readily available at academic institutions. Although the training of nonspecialized internists and general practitioners differs (6 or 5 years in internal medicine vs. 3 [before 2006] or 5 years [after 2006] in various fields of medicine including internal medicine), they are both entitled to provide primary care. For the purpose of this study, non‐oncological internists and general practitioners were therefore combined in one group (subsequently referred to as primary care physicians).

The prospective part of the ABC study, which is the subject of this publication, was based on information provided by the follow‐up physicians of the patients surveyed in the retrospective part. Its main goals were to characterize the three provider types and their patients, specify their information sources, explore their expectations of follow‐up care, and compare their performance with respect to health‐related outcomes (e.g., relapse detection, secondary disease prevention and detection), resource use (e.g., laboratory tests, imaging), and their patients' quality of life.

## 2 METHODS

### 2.1 Eligibility

The patient eligibility criteria have been previously described.^30^ In brief, patients ≥18 years who had been diagnosed with and/or treated for a hematological malignancy at the University Hospital of Essen were eligible, if the interval between study inclusion and date of diagnosis (for untreated patients) or end of last treatment (for primary disease or relapse) was ≥3 years. In patients receiving continuous oral medication or low dose maintenance therapy after intensive induction, eligibility started 3 years after treatment initiation or end of induction, respectively. The 3‐year starting point of the study was chosen because relapse tends to occur early in hematological malignancies, mainly within the first 2 or 3 years.^31^, ^32^, ^33^ Because of its poor prognosis most patients with early relapse are not candidates for long‐term follow‐up care. Conditions included monoclonal gammopathy of undetermined significance (MGUS), multiple myeloma (MM), indolent non‐Hodgkin lymphoma including chronic lymphocytic leukemia (iNHL/CLL), myelodysplastic syndrome (MDS), myeloproliferative neoplasms including chronic myeloid leukemia (MPN/CML), aggressive non‐Hodgkin or Hodgkin's lymphoma (aNHL/HL), and acute myeloid or acute lymphoblastic leukemia (AML/ALL). Irrespective of the underlying disease, patients with a history of allogeneic transplantation were allocated to a separate group (AlloTx), because health issues arising ≥3 years after transplantation are more likely to be related to the procedure than to the disease.^30^ Patients were categorized according to the above‐mentioned disease groups and the three follow‐up institutions identified in the retrospective part of the study.^30^

### 2.2 Study design

The observational study was approved by the ethics committee of the University of Duisburg‐Essen (February 17, 2014; no. 14‐5692‐BO). All participating patients and physicians gave written informed consent.

In the retrospective part, the patients were asked to name the physicians providing follow‐up care and specify the date of their next scheduled follow‐up visit. Their quality of life was assessed using the EORTC QLQ C‐30 questionnaire (restricted to the broad domains “global health”, “functioning” [physical, role, cognitive, emotional, and social combined] and “symptoms”) and the Hospital Anxiety and Depression Scale (HADS) questionnaires. The quality‐of‐life assessment in the prospective part was independent of the assessment in the retrospective part.^30^

The physician questionnaire was developed in three steps by the authors. The first draft was designed by JB, modified by UD, and then discussed among all authors. As a result, the questionnaire was split into three individual documents fulfilling different goals. In the final step, the questionnaires were completed by independent physicians of the Department of Hematology which led to minor changes in wording. The goals of the three documents were to (1) compare the characteristics of participating and nonparticipating physicians (questionnaire 1 [participation form] to be completed by both participating and nonparticipating physicians), (2) compare the attitudes of participating physicians from the three types of follow‐up institutions towards follow‐up care for a particular patient (questionnaire 2 [general aspects], only to be completed by participating physicians), and (3) compare the measures taken during follow‐up visits (questionnaire 3 [visit‐specific], to be completed by participating physicians, if their patient's visit fell within the 18‐month study period). If a patient had more than one follow‐up visit during the study period, questionnaire 3 was used repeatedly. The original questionnaires are provided in the Supporting Information. In brief, the questionnaires addressed the following questions:

1. The 2‐page participation form included questions concerning the physicians' age, the year that they obtained their medical license, their medical specialty, current professional position, and follow‐up care guidelines used.
2. The 3‐page general aspects questionnaire covered the exact blood‐cancer diagnosis of a particular patient as known by the follow‐up physician, comorbidities, frequency of follow‐up visits, and the physician‐perceived importance of follow‐up care for this patient.
3. The 5‐page visit‐specific questionnaire ensured the prospective documentation of follow‐up visits. Events to be documented included disease detection (relapse, second primary malignancy, any other new disease possibly related to the hematological malignancy or its treatment), counseling for disease prevention (cancer and cardiovascular screening programs, vaccinations), other consultation topics, physical examination, laboratory investigations, imaging, and organ function tests. Laboratory tests were divided into basic parameters (blood count including differential; basic plasma coagulation tests; serum lactate dehydrogenase, electrolytes, kidney and liver function parameters, total protein; urinalysis) and extensive investigations (lymphocyte subpopulations; serum protein electrophoresis, immunoglobulins, hormones, vitamins, tumor markers, iron metabolism; molecular analyses). The physicians were also asked to specify the date of the next visit which was documented in the same way if it fell within the 18‐month study period.

The physicians were contacted by mail and asked to participate in the study and complete the questionnaires. If they did not respond within 4–6 weeks, they were contacted by mail again, and if they also failed to respond to the second letter, they were contacted by phone.^34^ Each questionnaire was rewarded with 15 €. Monetary incentives have previously been shown to increase physician response rates in medical surveys.^35^

### 2.3 Statistical analysis

Frequencies are presented as numbers and compared using the chi^2^ test. Unless otherwise stated, percentages refer to the total number of patients, that is, they are not corrected for missing data. Continuous data are presented as median, first and third quartile (interquartile range, IQR), compared using the Kruskal‐Wallis test, and graphically displayed as box‐whisker plots, diamonds representing means. All analyses were exploratory, assuming statistical significance at *p* ≤ 0.05.

The quality‐of‐life scales were normalized to attain maximum power. Details of the procedure have been described before.^30^ The statistical analyses were performed using SAS (SAS version 9.4, SAS Institute Inc., Cary, NC, USA).

## 3 RESULTS

### 3.1 Patients

Of 2386 patients meeting the inclusion criteria, 1551 (65.0%) consented to participate in the study (Table 1). The characteristics of participating and nonparticipating patients have been reported elsewhere.^30^

**TABLE 1:** Patient characteristics.^30^

Total number of patients	1551
No allogeneic transplantation—number of patients (% of total)	997 (64.3%)
MGUS	19
MM	37
iNHL/CLL	264
MPN/CML	107
MDS	5
aNHL/HL	491
AML/ALL	74
Allogeneic transplantation^a^—number of patients (% of total)	554 (35.7%)
Age at study entry—years, median (range)	57.6 (23.0–91.2)
Time from diagnosis—years, median (range)	10.5 (3.0–40.7)
Time from last treatment^b^—years, median (range)	8.9 (3.0–36.0)
Male—number of patients (% of total)	841 (54.2%)
Female—number of patients (% of total)	710 (45.8%)
Follow‐up period—number of patients (% of total)	
Year 4–5	292 (18.8%)
Year 6–10	528 (34.1%)
Year >10	731 (47.1%)
Major follow‐up institution—number of patients (% of total)	
University Hospital of Essen (academic oncologists)	1045 (67.4%)
Community oncologists	231 (14.9%)
Primary care providers^c^	203 (13.1%)
No follow‐up	72 (4.6%)

Abbreviations: AML, acute myeloid leukemia; aNHL, aggressive non‐Hodgkin lymphoma; ALL, acute lymphoblastic leukemia; CLL, chronic lymphocytic leukemia; CML, chronic myeloid leukemia; iNHL, indolent non‐Hodgkin lymphoma; HL, Hodgkin lymphoma; MGUS, monoclonal gammopathy of undetermined significance; MM, multiple myeloma; MPN, myeloproliferative neoplasm; MDS, myelodysplastic syndrome.

^a^ Allogeneic transplantation for MM, 9; iNHL/CLL, 24; MPN/CML, 219; MDS, 40; aNHL/HL, 23; AML/ALL, 239.

^b^ Time from last treatment in 1279 treated patients (82.5% of total patients).

^c^ Follow‐up provided by non‐oncological internists (99 patients), general practitioners (94 patients), or others (10 patients).^30^

### 3.2 Characteristics of follow‐up physicians (questionnaire 1)

The patients named a total of 1070 physicians involved in follow‐up care. Seventy‐two patients named no follow‐up physician (abstention from follow‐up care), 729 named one, 706 named two, and 44 named three physicians. Of these, 223 were hematologists and medical oncologists (which is a single medical specialty in Germany; subsequently referred to as “oncologists”), 366 were internists (without specialization or specialized in fields other than hematology‐oncology), 386 were general practitioners, and 95 were specialists in other disciplines. Eighty‐eight physicians worked at the university hospital, 61 at other hospitals, 498 in individual private practice, and 423 in group private practice (Table 2).

**TABLE 2:** Participation of physicians in the ABC study.

	Number of physicians affected (% of total number of physicians named by patients)					Total
	Participation		No participation			
	Full documentation	Partial documentation	Refusal	Disregard	Retirement	
Medical specialty						
Oncologist	81 (36.4%)	38 (17.0%)	31 (13.9%)	71 (31.8%)	2 (0.9%)	223
Non‐oncological internist	66 (18.0%)	96 (26.3%)	92 (25.1%)	110 (30.1%)	2 (0.5%)	366
General practitioner	74 (19.2%)	107 (27.7%)	90 (23.3%)	110 (28.5%)	5 (1.3%)	386
Other discipline	5 (5.3%)	11 (11.6%)	24 (25.3%)	54 (56.8%)	1 (1.0%)	95
Location						
University Hospital Essen	28 (31.8%)	12 (13.6%)	10 (11.4%)	38 (43.2%)	0 (0.0%)	88
Other hospitals	11 (18.0%)	12 (19.7%)	17 (27.9%)	21 (34.4%)	0 (0.0%)	61
Individual private practice	77 (15.5%)	94 (18.9%)	130 (26.1%)	192 (38.5%)	5 (1.0%)	498
Group private practice	110 (26.0%)	134 (31.7%)	80 (18.9%)	94 (22.2%)	5 (1.2%)	423

**FIGURE 1:**
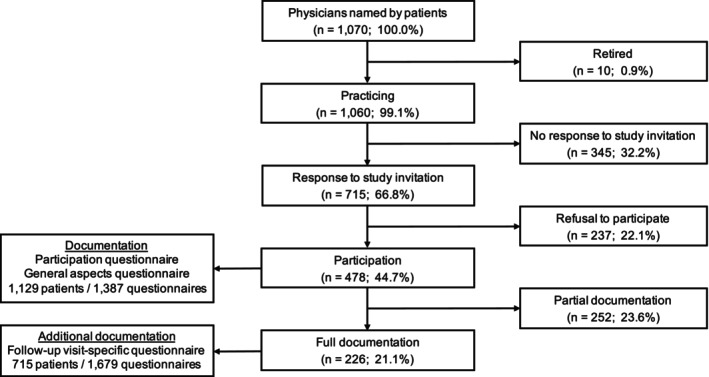
Flow diagram of physicians involved in follow‐up care (from being named by long‐term blood cancer survivors to participating in the ABC study and documenting follow‐up care). Full documentation means completion of all three physician questionnaires; partial documentation means completion of participation and general aspects questionnaires only.

Four hundred and seventy‐eight physicians consented to participate (44.7%). Reasons for nonparticipation included retirement, disregard of three invitations to participate, and refusal (Figure 1). Physicians consenting or refusing to participate were similar with regard to sex (male, 72.6 vs. 73.4%), age (median 61 [range, 30–74] vs. 56 [42–68] years), and work experience (20 [1–48] vs. 19 [3–36] years). Consent was highest among oncologists (119/223, 53.4%), intermediate among general practitioners (181/386, 46.9%) and nononcological internists (162/366, 44.3%), and lowest among specialists in other disciplines (16/95, 16.8%; *p* < 0.0001). Physicians in group private practice (244/423, 57.7%) were more likely to participate than physicians in other work environments (individual private practice, 171/498, 34.3%; university hospital, 40/88, 45.5%; other hospital, 23/61, 37.7%; *p* < 0.0001) (Table 2). Participation of physicians outside the university hospital was correlated with the number of follow‐up patients that they cared for (median, 1; range, 1–11), increasing from 31.6% for physicians caring for a single patient to 100% for those caring for ≥6 patients.

### 3.3 Follow‐up guidelines used by physicians (questionnaire 1)

Oncologists primarily relied on the concise “Onkopedia” guidelines^26^ of the German Society of Hematology and Medical Oncology (74.0%), closely followed by the more comprehensive guidelines of the Association of the Scientific Medical Societies in Germany (61.3%).^27^ Some oncologists (12.6%) also used international guidelines (Table S1). Non‐oncological internists (66.7%) and general practitioners (65.2%) primarily relied on information received from physicians previously caring for their patients. Reliance on knowledge acquired during postgraduate medical training was more common among oncologists (58.0%) and internists (51.9%) than among general practitioners (24.9%). The differences between the medical disciplines were statistically highly significant (Table S1; *p* < 0.0001 for all comparisons).

### 3.4 Physician‐perceived importance of follow‐up care (questionnaire 2)

A major result of the ABC study's retrospective part was the identification of three follow‐up care provider groups^30^: academic oncologists from the university hospital (40 of whom participated in the prospective part); community oncologists (84 participants) working in private practice (66 participants) or outpatient clinics (18 participants); and general practitioners (180 participants), non‐oncological internists (158 participants), and other physicians without specialization in oncology (16 participants) in private practice. Because of overlapping roles in the German health care system, the last‐named disciplines were combined in a single group (collectively referred to as “primary care physicians”).

The participating physicians returned 1387 questionnaires addressing general aspects of follow‐up care for 1129 of 1479 participating follow‐up patients (76.3%; academic oncologists, 857 questionnaires; community oncologists, 162; primary care physicians, 368). Eight hundred and seventy one patients were covered by a single questionnaire and 258 were covered by two separate questionnaires from different physicians.

All groups of physicians agreed that relapse detection was the most important goal of follow‐up care, followed by second primary malignancies, cardiovascular diseases, and infection. Polyneuropathy, psychosocial and fertility issues received lower scores (Table 3). As a rule, primary care physicians attributed higher importance to follow‐up care than oncologists did. This was true for all domains investigated (Table 3; *p* < 0.0001 for most comparisons) and confirmed when the analysis was restricted to patients who were covered by both an oncologist and a primary care physician (Figure S1; *p* < 0.0001 for all comparisons).

**TABLE 3:** Importance of individual aspects of follow‐up care as perceived by blood cancer survivors' physicians.

Detection of …	Number of votes/number of patients evaluated (%)			*p*
	Academic oncologists	Community oncologists	Primary care physicians	
Relapse				
Very important	446 / 787 (56.7%)	89 / 131 (67.9%)	252 / 310 (81.3%)	<0.0001
Important	185 / 787 (23.5%)	30 / 131 (22.9%)	41 / 310 (13.2%)	
Less important	138 / 787 (17.5%)	9 / 131 (6.9%)	14 / 310 (4.5%)	
Unimportant	18 / 787 (2.3%)	3 / 131 (2.3%)	3 / 310 (1.0%)	
Secondary malignancy				
Very important	363 / 788 (46.1%)	71 / 138 (51.4%)	190 / 310 (61.3%)	<0.0001
Important	333 / 788 (42.1%)	60 / 138 (43.5%)	105 / 310 (33.9%)	
Less important	88 / 788 (11.2%)	7 / 138 (5.1%)	13 / 310 (4.2%)	
Unimportant	4 / 788 (0.5%)	0 / 138 (0.0%)	2 / 310 (0.6%)	
Cardiovascular disease				
Very important	247 / 788 (31.3%)	41 / 136 (30.2%)	155 / 310 (50.0%)	<0.0001
Important	428 / 788 (54.3%)	66 / 136 (48.5%)	123 / 310 (39.7%)	
Less important	103 / 788 (13.1%)	28 / 136 (20.6%)	31 / 310 (10.0%)	
Unimportant	10 / 788 (1.3%)	1 / 136 (0.7%)	1 / 310 (0.3%)	
Fertility issues				
Very important	32 / 788 (4.1%)	10 / 134 (7.5%)	34 / 308 (11.0%)	0.0010
Important	110 / 788 (14.0%)	14 / 134 (10.4%)	41 / 308 (13.3%)	
Less important	303 / 788 (38.4%)	43 / 134 (32.1%)	107 / 308 (34.8%)	
Unimportant	343 / 788 (43.5%)	67 / 134 (50.0%)	126 / 308 (40.9%)	
Infection				
Very important	245 / 787 (31.1%)	43 / 138 (31.1%)	131 / 311 (42.1%)	<0.0001
Important	271 / 787 (34.5%)	64 / 138 (46.4%)	118 / 311 (37.9%)	
Less important	208 / 787 (26.4%)	28 / 138 (20.3%)	55 / 311 (17.7%)	
Unimportant	63 / 787 (8.0%)	3 / 138 (2.2%)	7 / 311 (2.3%)	
Polyneuropathy				
Very important	54 / 787 (6.9%)	25 / 138 (18.1%)	96 / 308 (31.2%)	<0.0001
Important	314 / 787 (39.9%)	65 / 138 (47.1%)	146 / 308 (47.4%)	
Less important	328 / 787 (41.7%)	40 / 138 (29.0%)	59 / 308 (19.1%)	
Unimportant	91 / 787 (11.5%)	8 / 138 (5.8%)	7 / 308 (2.3%)	
Psychosocial issues				
Very important	86 / 773 (11.1%)	33 / 134 (24.6%)	124 / 306 (40.5%)	<0.0001
Important	365 / 773 (47.2%)	81 / 134 (60.5%)	137 / 306 (44.8%)	
Less important	261 / 773 (33.8%)	18 / 134 (13.4%)	40 / 306 (13.1%)	
Unimportant	61 / 773 (7.9%)	2 / 134 (1.5%)	5 / 306 (1.6%)	
Counseling for …				
Cancer screening				
Very important	187 / 789 (23.7%)	23 / 134 (17.2%)	158 / 304 (52.0%)	<0.0001
Important	410 / 789 (52.0%)	76 / 134 (56.7%)	136 / 304 (44.7%)	
Less important	144 / 789 (18.2%)	29 / 134 (21.6%)	7 / 304 (2.3%)	
Unimportant	48 / 789 (6.1%)	6 / 134 (4.5%)	3 / 304 (1.0%)	
Cardiovascular risk factors				
Very important	219 / 787 (27.8%)	25 / 134 (18.7%)	164 / 320 (51.3%)	<0.0001
Important	406 / 787 (51.6%)	72 / 134 (53.7%)	136 / 320 (42.5%)	
Less important	141 / 787 (17.9%)	30 / 134 (22.4%)	18 / 320 (5.6%)	
Unimportant	21 / 787 (2.7%)	7 / 134 (5.2%)	2 / 320 (0.6%)	
Vaccination				
Very important	187 / 790 (23.7%)	21 / 134 (15.7%)	156 / 310 (50.3%)	<0.0001
Important	204 / 790 (25.8%)	68 / 134 (50.7%)	139 / 310 (44.9%)	
Less important	359 / 790 (45.4%)	37 / 134 (27.6%)	14 / 310 (4.5%)	
Unimportant	40 / 790 (5.1%)	8 / 134 (6.0%)	1 / 310 (0.3%)	

*Note*: *p*, chi^2^ test.

The physicians were also asked to rate the importance of preventive care. Counseling for cancer screening, cardiovascular risk factors, and vaccination was considered more important by primary care physicians than by oncologists (Table 3; p < 0.0001 for all comparisons).

### 3.5 Prospective documentation of follow‐up visits (questionnaire 3)

Based on the patients' ranking, each patient was allocated to a single institution predominantly responsible for follow‐up care (Table 1). The major follow‐up institutions returned 1679 visit‐specific questionnaires, covering 715 of 1479 follow‐up patients (48.3%) from all disease groups except MDS (Table 4). Coverage was highest at the university hospital (574 of 1045 follow‐up patients, 54.9%), followed by community oncologists (90/231, 39.0%) and primary care physicians (51/203, 25.1%). The disease spectrum differed at the three institutions (*p* < 0.0001). The largest groups in relation to total group size were AlloTx and iNHL/CLL for academic oncologists, MGUS, MM, and MPN/CML for community oncologists, and AML/ALL and aNHL/HL for primary care physicians (Table 4).

**TABLE 4:** Prospective documentation of follow‐up visits—institutions, disease groups, patients.

Disease group	Number of patients documented by institution / total number of patients documented per disease group (%)		
	Academic oncologists	Community oncologists	Primary care physicians
MGUS	0 / 4 (0.0%)	4 / 4 (100%)	0 / 4 (0.0%)
MM	11 / 18 (61.1%)	7 / 18 (39.9%)	0 / 18 (0.0%)
iNHL/CLL	99 / 123 (80.5%)	15 / 123 (12.2%)	9 / 123 (7.3%)
MPN/CML	37 / 55 (67.3%)	17 / 55 (30.9%)	1 / 55 (1.8%)
aNHL/HL	105 / 145 (72.4%)	25 / 145 (17.2%)	15 / 145 (10.4%)
AML/ALL	18 / 29 (62.1%)	3 / 29 (10.3%)	8 / 29 (27.6%)
AlloTx	304 / 341 (89.1%)	19 / 341 (5.6%)	18 / 341 (5.3%)
Total	574 / 715 (80.3%)	90 / 715 (12.6%)	51 / 715 (7.1%)

*Note*: chi^2^ test, *p* < 0.0001.

Abbreviations: AML, acute myeloid leukemia; ALL, acute lymphoblastic leukemia; AlloTx, allogeneic transplantation; CLL, chronic lymphocytic leukemia; CML, chronic myeloid leukemia; HL, Hodgkin lymphoma; iNHL, indolent non‐Hodgkin lymphoma; MGUS, monoclonal gammopathy of undetermined significance; MM, multiple myeloma; MPN, myeloproliferative neoplasm; NHL, aggressive non‐Hodgkin lymphoma.

The average number of documented visits was 2 (range, 1–6). Items listed in the questionnaire were regarded as addressed, if they were documented at least once during the study period.

#### 3.5.1 Relapse, second primary malignancy, and other diseases

During the 18‐month study period, the physicians reported 58 blood cancer relapses (8.1% of 715 patients), 25 second primary malignancies (3.5%), 22 other noninfectious new diseases (3.1%), 37 acute infections (5.2%), and 47 cancer‐ or cancer‐therapy‐related chronic diseases (6.6%). Except for acute infections, there were no significant differences in the disease detection rates among the three types of follow‐up institutions (Table 5). The diseases reported by the three institutions in the seven blood‐cancer disease groups are detailed in Table S2.

**TABLE 5:** Events documented at follow‐up visits during the 18‐months prospective study period.

Events documented	Number of patients affected/total number of patients documented by institution			*p*
	Academic oncologists	Community oncologists	Primary care physicians	
Diseases				
Relapse or progression	48 / 574 (8.4%)	9 / 90 (10.0%)	1 / 51 (2.0%)	0.2157
Second primary malignancy	19 / 574 (3.3%)	2 / 90 (2.2%)	4 / 51 (7.8%)	0.1875
Other noninfectious new diseases	19 / 574 (3.3%)	0 / 90 (0.0%)	3 / 51 (5.9%)	0.1160
Acute infections	31 / 574 (5.4%)	0 / 90 (0.0%)	6 / 51 (11.8%)	0.0087
Chronic diseases	39 / 574 (6.8%)	7 / 90 (7.8%)	1 / 51 (2.0%)	0.3633
Counseling for disease prevention				
Cancer screening	283 / 574 (49.3%)	24 / 90 (26.7%)	28 / 51 (54.9%)	0.0002
Cardiovascular risk factors	241 / 574 (42.0%)	28 / 90 (31.1%)	33 / 51 (64.7%)	0.0005
Vaccination	258 / 574 (44.9%)	14 / 90 (15.6%)	35 / 51 (68.6%)	<0.0001
Consultation topics				
Psychosocial issues	96 / 574 (16.7%)	18 / 90 (20.0%)	16 / 51 (31.4%)	0.0304
Sexuality	19 / 574 (3.3%)	6 / 90 (6.7%)	8 / 51 (15.7%)	0.0002
Fertility^a^	30 / 574 (5.2%)	3 / 90 (3.3%)	1 / 51 (2.0%)	0.4578
Infection	321 / 574 (55.9%)	38 / 90 (42.2%)	13 / 51 (25.5%)	<0.0001
Polyneuropathy	122 / 574 (21.3%)	24 / 90 (26.7%)	17 / 51 (33.3%)	0.0926
Physical examination				
General physical examination	483 / 574 (84.1%)	56 / 90 (62.2%)	41 / 51 (80.4%)	<0.0001
Blood pressure measurement	361 / 574 (62.9%)	18 / 90 (20.0%)	39 / 51 (76.5%)	<0.0001
Laboratory investigations				
Basic laboratory tests	513 / 574 (89.4%)	62 / 90 (68.9%)	40 / 51 (78.4%)	<0.0001
Extensive laboratory tests	366 / 574 (63.8%)	35 / 90 (38.9%)	27 / 51 (52.9%)	<0.0001
Imaging				
Abdominal ultrasonography	178 / 574 (31.0%)	35 / 90 (38.9%)	27 / 51 (52.9%)	0.0033
Lymph node ultrasonography	8 / 574 (1.4%)	13 / 90 (14.4%)	3 / 51 (5.9%)	<0.0001
Chest X‐ray	78 / 574 (13.6%)	2 / 90 (2.2%)	6 / 51 (11.8%)	0.0086
Skeletal X‐ray	11 / 574 (1.9%)	0 / 90 (0.0%)	2 / 51 (3.9%)	0.2274
Computed tomography	14 / 574 (2.4%)	6 / 90 (6.7%)	5 / 51 (9.8%)	0.0050
Magnetic resonance imaging	12 / 574 (2.1%)	4 / 90 (4.4%)	3 / 51 (5.9%)	0.1441
Organ function tests				
Electrocardiography	25 / 574 (4.4%)	3 / 90 (3.3%)	20 / 51 (39.2%)	<0.0001
Echocardiography	3 / 574 (0.5%)	0 / 90 (0.0%)	4 / 51 (7.8%)	<0.0001

*Note*: *p*, chi^2^ test.

^a^ Twenty‐one of 34 consultations covering fertility issues (61.8%) were performed in patients below age 50.

Relapse or progression was most frequent in MM (5 of 18 patients, 27.8%), iNHL/CLL (31/123, 25.2%), and MGUS (1/4 [progression to lymphoplasmacytic lymphoma], 25.0%) and less frequent in AML/ALL (2/29, 6.9%), MPN/CML (3/55, 5.4%), aNHL/HL (6/145, 4.1%), and AlloTx (10/341, 2.9%; *p* < 0.0001). While academic oncologists and community oncologists reported relapses in each of the blood‐cancer disease groups documented, primary care physicians recorded a relapse only in iNHL/CLL (Table S2).

Second primary malignancies were most frequent in iNHL/CLL (7/123, 5.7%) and AlloTx (15/341, 4.4%), rare in MPN/CML (1/55, 1.8%) and aNHL/HL (2/145, 1.4%), and not observed in MGUS, MM, and AML/ALL (*p* = 0.3386). They included one squamous and 13 basal cell carcinomas of the skin (iNHL/CLL, 5; MPN/CML, 1; AlloTx, 8), one anal, one esophageal and two oral squamous cell carcinomas (all AlloTx), two pancreatic cancers, and one case each of follicular lymphoma, diffuse large B‐cell lymphoma, breast cancer, liver cancer, and cancer of unknown primary. Academic oncologists observed second primary malignancies in four blood‐cancer disease groups (iNHL/CLL, MPN/CML, aNHL/HL, AlloTx), primary care physicians in three (iNHL/CLL, aNHL/HL, AlloTx), and community oncologists in one (AlloTx) (Table S2).

Other noninfectious new diseases were most frequently recorded in MPN/CML (3/55, 5.5%), followed by AlloTx (13/341, 3.8%), iNHL/CLL (3/123, 2.4%), and aNHL/HL (3/145, 2.1%; no cases in MGUS, MM, and AML/ALL; *p* = 0.6846). Chronic blood cancer‐ or treatment‐related diseases predominated in Allo‐Tx (39/341, 11.4%), followed by iNHL/CLL (5/123, 4.1%), MPN/CML (1/55, 1.8%), and aNHL/HL (2/145, 1.4%; no cases in MGUS, MM, and AML/ALL; *p* = 0.0002). Acute or chronic complications restricted to AlloTx included graft‐versus‐host disease (*n* = 32), femoral head necrosis (*n* = 4), depression (*n* = 2), and kidney failure (*n* = 1). Cutaneous ulcers (*n* = 2) were exclusively reported in MPN/CML. Diseases occurring in several blood cancer groups included cardiovascular complications (*n* = 10), polyneuropathy (*n* = 6), phlebothrombosis (*n* = 4), osteoporosis (*n* = 3), fatigue (*n* = 3), and cataract (*n* = 2). Although all three follow‐up institutions cared for a substantial number of AlloTx patients, chronic transplantation‐related sequelae, for example, graft‐versus‐host disease, were only reported by academic oncologists and community oncologists (Table S2).

Acute infections were most often recorded in iNHL/CLL (10/123, 8.1%) and AlloTx (23/341, 6.7%), followed by MM (1/18, 5.6%), MPN/CML (2/55; 3.6%), and aNHL/HL (1/145, 0.7%; no cases in MGUS and AML/ALL; *p* = 0.0638) (Table S2). Acute infections were significantly more often reported by primary care physicians (11.8%) and academic oncologists (5.4%) than by community oncologists (0.0%; *p* = 0.0087) (Table 5).

#### 3.5.2 Counseling for disease prevention

Counseling for disease prevention was more extensively done by primary care physicians than by academic oncologists or community oncologists (Table 5). The differences were statistically significant for cancer screening (54.9% vs. 49.3% vs. 26.7%; *p* = 0.0002), cardiovascular risk factors (64.7% vs. 42.0% vs. 31.1%; *p* = 0.0005), and vaccination (68.6% vs. 44.9% vs. 15.6%; p < 0.0001).

#### 3.5.3 Consultation topics and physical examination

Primary care physicians significantly more often addressed questions related to psychosocial issues and sexuality than academic oncologists or community oncologists did (psychosocial issues, 31.4% vs. 16.7% vs. 20.0%, *p* = 0.0304; sexuality, 15.7% vs. 3.3% vs. 6.7%, *p* = 0.0002). The converse was true for infections (25.5% vs. 55.9% vs. 42.2%; *p* < 0.0001). Physical examination was more often performed by academic oncologists (84.1%) and primary care physicians (80.4%) than by community oncologists (62.2%; *p* < 0.0001) (Table 5).

#### 3.5.4 Laboratory investigations

Laboratory tests were more frequently ordered by academic oncologists than by community oncologists or primary care physicians (basic tests, 89.4% vs. 68.9% vs. 78.4%, *p* < 0.0001; extensive tests, 63.8% vs. 38.9% vs. 52.9%, *p* < 0.0001) (Table 5). Extensive tests were more often performed in AlloTx (269/341, 78.9%), MM (14/18, 77.8%), and iNHL/CLL (70/123, 56.9%) than in MPN/CML (18/55, 32.7%), aNHL/HL (43/145, 29.7%), AML/ALL (8/29, 27.6%), or MGUS (1/4, 25.0%; *p* < 0.0001). Their frequency remained constant over time (year 4–5, 107 of 182 patients, 58.8%; years 6–10, 195/330, 58.8%; year >10, 127/203; 62.6%; *p* = 0.6772).

#### 3.5.5 Imaging and organ function tests

Imaging was more often ordered by primary care physicians than by academic oncologists or community oncologists (90.2 vs. 66.7 vs. 52.4 investigations per 100 patients; *p* < 0.0001). The same was true for electrocardiography and echocardiography (Table 5). Other organ function tests were rarely performed (data not shown).

### 3.6 Quality of life of patients visiting different follow‐up institutions

**FIGURE 2:**
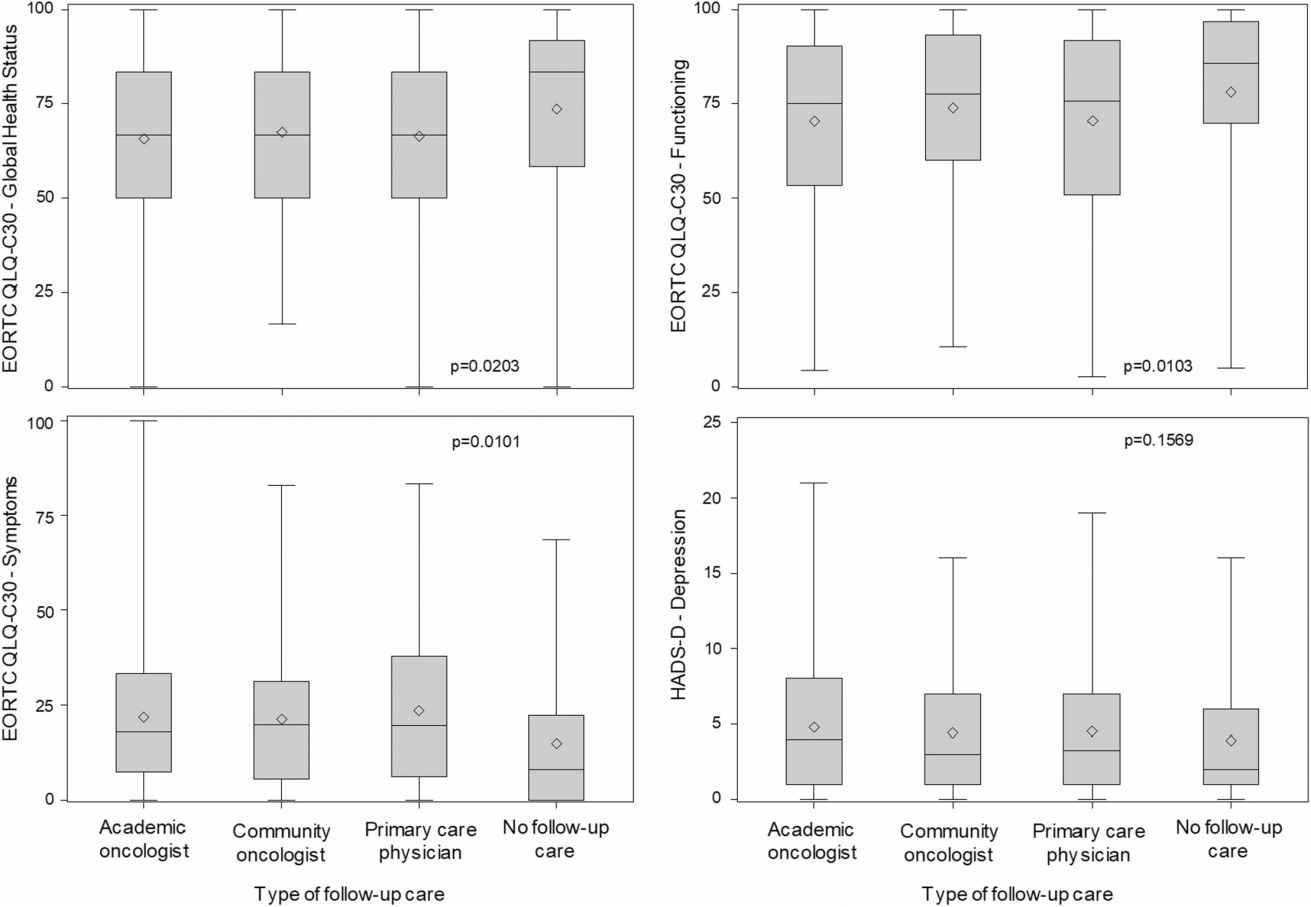
Quality‐of‐life in long‐term blood cancer survivors in relation to the institution providing follow‐up care, as assessed by the EORTC QLQ‐C30 (global health status, functioning, symptoms) and HADS questionnaires (depression). The results of the HADS anxiety scale (not shown) were similar to those of the depression scale. Academic oncologists, 955 patients; community oncologists, 210 patients; primary care providers, 183 patients; no follow‐up, 59 patients).

The quality‐of‐life assessment included 1348 patients on follow‐up care and 59 patients not undergoing follow‐up care. While quality of life was significantly better in patients forgoing follow‐up care than in patients utilizing it, there were no statistically significant differences between the three follow‐up institutions (Figure 2).

## 4 DISCUSSION

The major results of the ABC study's prospective part are the following: First, less than half of follow‐up physicians consented to participate in the study. Second, most blood cancer survivors with a consenting follow‐up physician received care at the university hospital, with only a minority being cared for by community oncologists or primary care physicians. Third, the disease spectrum differed among follow‐up institutions. Fourth, although physicians of different disciplines used different follow‐up guidelines, they agreed on the goals of follow‐up care. Finally, relapse and secondary disease detection rates and the patients' reports about their quality of life were similar at all follow‐up institutions, but there were significant differences in other domains.

Compared to other questionnaire‐based survivorship studies and despite a monetary incentive,^35^ the participation rate among follow‐up physicians appeared low (45% vs. 62%–76% in previous studies).^36^, ^37^, ^38^ This may have been related to the fact that the patients were asked to name one or more follow‐up physicians. In general, only the first‐named physician had a vital role in follow‐up care. Therefore, patient coverage by at least one physician was much higher (76%) than overall physician participation (45%). Unfortunately, considerable attrition occurred between the general aspects questionnaire (covering 76% of participating patients) and the visit‐specific questionnaire (covering only 48%), in particular in patients treated outside the university hospital. This may have been related to the size of the visit‐specific questionnaire. Survey length is negatively correlated with response rate.^35^

Overall, 80% of patients with fully‐documented follow‐up visits were treated by academic oncologists, 13% by community oncologists, and 7% by primary care physicians. The disease spectrum differed at the three types of follow‐up institutions. Patients at high risk of relapse (iNHL/CLL) or treatment‐related long‐term problems (AlloTx) were predominantly followed up at the university hospital (80–90% of all iNHL/CLL or AlloTx patients). By contrast, patients in stable condition with diseases requiring continuous monitoring with or without oral maintenance therapy (MGUS, MM, MPN/CML) were frequently seen by community oncologists (30–100% of such patients). Survivors seen by primary care physicians most often had a history of a curable disease and were in stable long‐term remission (10–30% of aNHL/HL and AML/ALL patients). The few iNHL/CLL or AlloTx patients followed up by primary care physicians appeared to be a particularly favorable subgroup, because chronic treatment‐related disease states were not reported and relapses were rare.

The major information sources used for follow‐up care were national guidelines^26^, ^27^ for oncologists, and recommendations by other physicians for primary care providers. Ideally, such recommendations would be included in an individualized survivorship care plan accessible for all involved physicians.^3^, ^5^, ^16^ The implementation of survivorship care plans, however, has been difficult because of time constraints, responsibility and reimbursement issues, and a paucity of data demonstrating its positive impact on patient outcome.^3^ In Germany, communication between hospitals and private practices is mainly based on discharge letters that may or may not contain recommendations for follow‐up care. Both in Germany^39^ and other countries,^40^, ^41^ primary care physicians have complained about insufficient information from cancer hospitals. This may have contributed to the low number of primary care providers participating in the ABC study.

Despite the use of different information sources, relapse detection, second primary malignancies, and cardiovascular diseases were consistently viewed as the most important goals of follow‐up care. With regard to these endpoints, the performance of the three institutions appeared similar. The resources used, however, differed. While laboratory investigations, crucial and recommended for all types of blood cancer,^26^, ^27^ were most extensively performed at the university hospital, imaging was most often ordered by primary care physicians. The role of imaging in blood cancer follow‐up care is limited, in particular in survivors with a history of leukemia and lymphoma^29^, ^42^ that were disproportionately often seen by primary care physicians. National and international guidelines explicitly discourage the use of routine surveillance scans in lymphoma.^26^, ^27^, ^29^ Primary care providers have previously been reported to have an incommensurate tendency to order imaging tests for cancer survivors. Explanations included defensive medicine, reimbursement incentives, and uncertainty about guidelines.^38^

Follow‐up care includes management of psychosocial consequences, promotion of a healthy life style, and disease prevention.^2^, ^3^, ^4^, ^43^, ^44^ These needs were better addressed by primary care physicians than by oncologists. Differences were particularly pronounced in counseling for disease prevention. Our findings are consistent with a report from the USA where cancer survivors expected their follow‐up physicians to provide preventive health care irrespective of medical qualification. While most general practitioners agreed to provide preventive care, only a minority of oncologists considered screening for other cancers their responsibility.^37^

Although the patients' needs appeared to be more comprehensively addressed by primary care physicians than by oncologists, one cannot conclude from our data that follow‐up care should be shifted to the former. First, the proportion of patients seen by primary care physicians was small, decreasing from 13.7% in the retrospective part of the study^30^ to 7.1% in the prospective part. Second, the disease spectrum differed in different follow‐up institutions. While survivors seen at the university hospital tended to be at high risk for relapse or late adverse events, most survivors seen by primary care physicians had a history of a curable disease and were in stable remission. Third, primary care providers participating in the ABC study were likely a selection of physicians with exceptional skills and motivations. In many countries, primary care physicians feel insufficiently trained, informed and equipped to provide comprehensive follow‐up care.^36^, ^40^, ^41^, ^45^ This is in line with our observation that, despite a multitude of primary care providers named as follow‐up physicians by their patients, only a minority played a dominant role. Those who did tended to rely on information received from others to provide adequate care. A recent report from Canada demonstrates that primary care providers can play a more important role in follow‐up care, if they receive adequate training.^45^ Whether this also applies to hematological diseases with a high risk of relapse and late complications, remains to be shown.

Apart from the physician selection bias mentioned above, our study has several other limitations. First, the number of follow‐up visits documented by physicians outside the university hospital was lower than expected. Second, the disease spectrum was wide, some disease groups were small, the duration of follow‐up varied, and the features of survivors seen by different groups of care providers differed, making comparisons difficult. Third, the original study population was restricted to patients from a single comprehensive cancer center. While the results are likely to be representative for many medical institutions in Germany, they may not apply to countries with other health care systems. Finally, the observational nature of our study did not allow us to verify the data provided. Strengths of our study are its large size and the prospective capture of events during a reasonably long follow‐up period.

Conclusions from the ABC study must be drawn with caution, because differences in the spectrum of hematological and secondary diseases among follow‐up institutions precluded an unbiased comparison. With this caveat, detection of relapse and secondary diseases was similar in all three follow‐up institutions. Psychosocial issues and preventive health care appeared to be better addressed by primary care physicians than by oncologists, while the converse was true for a judicious use of medical resources. For patients with curable diseases in stable remission, transfer of follow‐up care from oncologists to primary care providers seems feasible, provided the latter receive adequate information about the required procedures.

## AUTHOR CONTRIBUTIONS

**Hildegard Lax:** Data curation (supporting); formal analysis (supporting); methodology (supporting); visualization (supporting); writing – review and editing (supporting). **Julia Baum:** Data curation (lead); investigation (supporting); methodology (supporting); project administration (supporting); supervision (supporting); writing – review and editing (supporting). **Nils Lehmann:** Conceptualization (supporting); data curation (supporting); formal analysis (supporting); methodology (supporting); writing – review and editing (supporting). **Anja Merkel‐Jens:** Data curation (supporting); resources (supporting); writing – review and editing (supporting). **Dietrich Beelen:** Project administration (supporting); supervision (supporting); writing – review and editing (supporting). **Karl‐Heinz Jöckel:** Conceptualization (supporting); formal analysis (supporting); funding acquisition (supporting); methodology (supporting); project administration (supporting); supervision (supporting); writing – review and editing (supporting). **Ulrich Dührsen:** Conceptualization (lead); formal analysis (lead); funding acquisition (lead); investigation (lead); methodology (lead); project administration (lead); resources (lead); supervision (lead); visualization (lead); writing – original draft (lead); writing – review and editing (lead).

## FUNDING INFORMATION

The study was supported by the Federal Ministry of Education and Research of Germany (Bundesministerium für Bildung und Forschung, grant no. 01GY1341).

## CONFLICT OF INTEREST STATEMENT

The authors report no potential conflicts of interest relevant to this article.

## ETHICS STATEMENT

The study was performed in line with the principles of the Declaration of Helsinki. Approval was granted by the Ethics Committee of the University of Duisburg‐Essen (February 17, 2014; no. 14‐5692‐BO).

## Supporting information


Data S1.

## Data Availability

The datasets used and analyzed during this study are available from the corresponding author on reasonable request.
