# Supplementary material for: Patterns of follow‐up care in adult blood cancer survivors—Prospective evaluation of health‐related outcomes, resource use, and quality of life
Source: Cancer Med. 2024 Mar 29;13(7):e7095. doi: 10.1002/cam4.7095 (PMC10979186; doi:10.1002/cam4.7095)
Supplement: Supplementary file 1 — Data S1. [file CAM4-13-e7095-s001.pdf]

*Supporting information*

# Patterns of follow-up care in adult blood cancer survivors – prospective evaluation of health-related outcomes, resource use, and quality of life

Hildegard Lax,[1] Julia Baum,[2] Nils Lehmann,[1] Anja Merkel-Jens,[1]

Dietrich W. Beelen,[3] Karl-Heinz Jöckel,[1] Ulrich Dührsen[2]

[1] Institut für Medizinische Informatik, Biometrie und Epidemiologie, Universität Duisburg-Essen, Germany

[2] Klinik für Hämatologie, Universitätsklinikum Essen, Universität Duisburg-Essen, Germany

[3] Klinik für Knochenmarktransplantation, Universitätsklinikum Essen, Universität Duisburg-Essen, Germany

## Supporting information

**Supplementary Table 1**

**Supplementary Table 2**

**Supplementary Figure 1**

**Physician questionnaires**

Participation form

General aspects questionnaire

Visit-specific questionnaire

**Supplementary Table 1. Follow-up care guidelines used by physicians of different disciplines**

| Source | Number of physicians affected / number of physicians participating (%) | | | p |
|---|---|---|---|---|
| | **Oncologists** | **Non-oncological internists** | **General practitioners** | |
| ‘Onkopedia’ guidelines[a] (DGHO) | 88 / 119 (74.0%) | 17 / 162 (10.5%) | 15 / 181 (8.3%) | <0.0001 |
| ‘S3’ guidelines[b] (AWMF) | 73 / 119 (61.3%) | 59 / 162 (36.4%) | 55 / 181 (30.4%) | <0.0001 |
| International guidelines[c] | 15 / 119 (12.6%) | 0 / 162 (0.0%) | 0 / 181 (0.0%) | <0.0001 |
| Recommendation by previous physicians | 42 / 119 (35.3%) | 108 / 162 (66.7%) | 118 / 181 (65.2%) | <0.0001 |
| Postgraduate medical training | 69 / 119 (58.0%) | 84 / 162 (51.9%) | 45 / 181 (24.9%) | <0.0001 |

DGHO, German Society of Haematology and Medical Oncology; AWMF, Association of the Scientific Medical Societies; p, $chi^2$ test

[a] Onkopedia Leitlinien (established in 2010), Deutsche Gesellschaft für Hämatologie und Medizinische Onkologie (DGHO; German Society of Haematology and Medical Oncology). Accessed May 7, 2023. https://www.onkopedia.com/de/onkopedia/guidelines

[b] AWMF Leitlinien (established in 1995), Arbeitsgemeinschaft der Wissenschaftlichen Medizinischen Fachgesellschaften (AWMF; Association of the Scientific Medical Societies in Germany). Accessed May 7, 2023. https://register.awmf.org/de/leitlinien/aktuelle-leitlinien

[c] Guidelines of the European Society for Medical Oncology (ESMO), 10 oncologists; National Comprehensive Cancer Network (NCCN), 7 oncologists; American Society of Medical Oncology (ASCO), 4 oncologists

**Supplementary Table 2. Diseases documented at follow-up visits during the prospective 18-month study period**

| Diseases documented | Academic oncologists | | Community oncologists | | Primary care physicians | |
|---|---|---|---|---|---|---|
| | Type (number) | TNP | Type (number) | TNP | Type (number) | TNP |
| **Relapse or progression** | | | | | | |
| MGUS | NA | 0 | (1) | 4 | NA | 0 |
| MM | (4) | 11 | (1) | 7 | NA | 0 |
| iNHL/CLL | (28) | 99 | (2) | 15 | (1) | 9 |
| MPN/CML | (2) | 37 | (1) | 17 | (0) | 1 |
| aNHL/HL | (5) | 105 | (1) | 25 | (0) | 15 |
| AML/ALL | (1) | 18 | (1) | 3 | (0) | 8 |
| AlloTx | (8) | 304 | (2) | 19 | (0) | 18 |
| **Second primary malignancy** | | | | | | |
| MGUS | NA | 0 | 0 | 4 | NA | 0 |
| MM | 0 | 11 | 0 | 7 | NA | 0 |
| iNHL/CLL | BCC (4), breast cancer (1), pancreatic cancer (1) | 99 | 0 | 15 | BCC (1) | 9 |
| MPN/CML | BCC (1) | 37 | 0 | 17 | 0 | 1 |
| aNHL/HL | Follicular lymphoma (1) | 105 | 0 | 25 | Pancreatic cancer (1) | 15 |
| AML/ALL | 0 | 18 | 0 | 3 | 0 | 8 |
| AlloTx | BCC (5), cutaneous SCC (1), oral SCC (1), anal SCC (1), esophageal SCC (1), liver cancer (1), diffuse large B-cell lymphoma (1) | 304 | Cutaneous SCC (1), oral SCC (1) | 19 | BCC (1), CUP (1) | 18 |

| | | | | | | |
|---|---|---|---|---|---|---|
| **Other non-infectious new diseases** | | | | | | |
| MGUS | NA | 0 | 0 | 4 | NA | 0 |
| MM | 0 | 11 | 0 | 7 | NA | 0 |
| iNHL/CLL | Heart failure (1), phlebothrombosis (1) | 99 | 0 | 15 | Carotid artery stenosis (1) | 9 |
| MPN/CML | Heart failure (1), cutaneous ulcer (2) | 37 | 0 | 17 | 0 | 1 |
| aNHL/HL | Carotid artery stenosis (1), phlebothrombosis (1), cataract (1) | 105 | 0 | 25 | 0 | 15 |
| AML/ALL | 0 | 18 | 0 | 3 | 0 | 8 |
| AlloTx | Heart failure (2), myocardial infarction (1), atrial fibrillation (1), phlebothrombosis (1), renal failure (1), femoral head necrosis (4), cataract (1) | 304 | 0 | 19 | Myocardial infarction (1), phlebothrombosis (1) | 18 |
| **Acute infections** | | | | | | |
| MGUS | NA | 0 | 0 | 4 | NA | 0 |
| MM | Septicemia (1) | 11 | 0 | 7 | NA | 0 |
| iNHL/CLL | Upper airway infection (3), pneumonia (1), oral thrush (1), gastrointestinal infection (2), septicemia (1), shingles (1) | 99 | 0 | 15 | Shingles (1) | 9 |
| MPN/CML | Pneumonia (1), urinary tract infection (1) | 37 | 0 | 17 | 0 | 1 |
| aNHL/HL | Upper airway infection (1) | 105 | 0 | 25 | 0 | 15 |
| AML/ALL | 0 | 18 | 0 | 3 | 0 | 8 |

| | | | | | | |
|---|---|---|---|---|---|---|
| AlloTx | Upper airway infection (8), pneumonia (1), gastrointestinal infection (5), urinary tract infection (1), cutaneous abscess (1), erysipelas (1), shingles (1) | 304 | 0 | 19 | Upper airway infection (3), pneumonia (1), urinary tract infection (1) | 18 |
| **Chronic diseases** | | | | | | |
| MGUS | NA | 0 | 0 | 4 | NA | 0 |
| MM | 0 | 11 | 0 | 7 | NA | 0 |
| iNHL/CLL | PNP (2), fatigue (1) | 99 | PNP (1), osteoporosis (1) | 15 | 0 | 9 |
| MPN/CML | Fatigue (1) | 37 | 0 | 17 | 0 | 1 |
| aNHL/HL | Fatigue (1) | 105 | 0 | 25 | Osteoporosis (1) | 15 |
| AML/ALL | 0 | 18 | 0 | 3 | 0 | 8 |
| AlloTx | GvHD (28), PNP (3), osteoporosis (1), depression (2) | 304 | GvHD (4), lymphedema (1) | 19 | 0 | 18 |

BCC, basal cell carcinoma of the skin; CUP, cancer of unknown primary; GvHD, graft-versus-host disease; NA, not applicable (no patients documented); PNP, polyneuropathy; SCC, squamous cell carcinoma; TNP, total number of patients documented

**Supplementary Figure 1**

| A | | Primary care physician | | | |
|---|---|---|---|---|---|
| | | Very important | Important | Less important | Unimportant |
| Oncologist | Very important | 62 (46.9%) | 4 (3.0%) | 2 (1.5%) | 0 (0.0%) |
| | Important | 24 (18.2%) | 6 (4.5%) | 1 (0.8%) | 0 (0.0%) |
| | Less important | 24 (18.2%) | 2 (1.5%) | 1 (0.8%) | 1 (0.8%) |
| | Unimportant | 4 (3.0%) | 0 (0.0%) | 1 (0.8%) | 0 (0.0%) |

| B | | Primary care physician | | | |
|---|---|---|---|---|---|
| | | Very important | Important | Less important | Unimportant |
| Oncologist | Very important | 4 (3.1%) | 4 (3.1%) | 1 (0.8%) | 0 (0.0%) |
| | Important | 24 (18.6%) | 26 (20.2%) | 5 (3.9%) | 0 (0.0%) |
| | Less important | 26 (20.2%) | 23 (17.8%) | 4 (3.1%) | 0 (0.0%) |
| | Unimportant | 2 (1.5%) | 4 (3.1%) | 4 (3.1%) | 2 (1.5%) |

**Concordance in physician-perceived importance of follow-up goals within pairs of oncologists and primary care physicians caring for the same blood cancer patient.** A. Relapse detection – 52.2% concordance in 132 patients, higher importance perceived by primary care physicians (chi$^2$ test, $p<0.0001$). B. Detection of psychosocial issues – 27.9% concordance in 129 patients, higher importance perceived by primary care physicians (chi$^2$ test, $p<0.0001$). In case of complete agreement, the percentages in the grey-shaded boxes would add up to 100%.

Participation form

Universitätsklinikum Essen

westdeutsches tumorzentrum

**Klinik für Hämatologie**
Dir.: Prof. Dr. med. U. Dührsen

# ABC-Studie

GEFÖRDERT VOM
Bundesministerium für Bildung und Forschung

## Teilnahmeerklärung

**Nachsorgemuster bei erwachsenen Patientinnen und Patienten mit Blutkrebs und Blutkrebsvorstufen: Einfluss auf gesundheitsbezogene Endpunkte, Lebensqualität und Ressourcennutzung**

Ihr Name, Vorname: ______________________________

Geburtsjahr: ______________________________

Geschlecht: ☐ weiblich ☐ männlich

Name der Praxis/ Klinik: ______________________________
(bitte in Druckbuchstaben)

☐ Fachärztin/ Facharzt ☐ Ärztin/ Arzt i. Weiterbildung

Fachgebiet: ______________________________

Jahr der Anerkennung ______________________________

Zusatzbezeichnung: ______________________________

Ich bin tätig:

☐ in einem Krankenhaus/ einer Klinikambulanz
☐ selbständig in eigener Praxis
☐ angestellt in einer Praxis
☐ Partner einer Gemeinschaftspraxis
☐ in einer Praxisgemeinschaft
☐ in einem MVZ
☐ sonstiges: ______________________________

Approbationsjahr: ______________________________

Welche Informationsquellen über die Gestaltung der Nachsorgeuntersuchungen nutzen Sie?
(Mehrfachnennung möglich)

☐ National oder international verfügbare Leitlinien:
- ☐ DGHO-Leitlinien (Onkopedia)
- ☐ S3-Leitlinien
- ☐ andere Leitlinien, und zwar: ______________________________

☐ Erfahrungen aus der Facharztweiterbildung
☐ Empfehlungen des vorbehandelnden Arztes für den jeweiligen Patienten
☐ Empfehlungen in der Fachliteratur
☐ Sonstiges, ______________________________

Arzt-Nr

Participation form

Finden Sie die verfügbaren Informationen zur Gestaltung der Nachsorgeuntersuchungen ausreichend?

☐ Ja

☐ Nein, ich wünsche mir:

________________________________________

________________________________________

________________________________________

**Teilnahme an der ABC-Studie:**

☐ Ich nehme teil

☐ Ich möchte nicht teilnehmen, weil:

________________________________________

________________________________________

**Meine Kontodaten:**

| | |
|---|---|
| Kontoinhaber | ____________________ |
| ggf. Verwendungszweck | ____________________ |
| Kreditinstitut | ____________________ |
| IBAN | ____________________ |
| BIC | ____________________ |

________________ ____________________________

Ort, Datum Unterschrift + Praxisstempel

Arzt-Nr

Universitätsklinikum Essen

westdeutsches tumorzentrum

Klinik für Hämatologie
Dir.: Prof. Dr. med. U. Dührsen

**ABC-Studie**

GEFÖRDERT VOM
Bundesministerium für Bildung und Forschung

**Nachsorgemuster bei erwachsenen Patientinnen und Patienten mit Blutkrebs und Blutkrebsvorstufen: Einfluss auf gesundheitsbezogene Endpunkte, Lebensqualität und Ressourcennutzung**

# Arztfragebogen zur Nachsorge
# Allgemeine Aspekte

**Ihre Patientin/Ihr Patient:**

*Sind die Angaben zu Ihrer Patientin/Ihrem Patienten korrekt? Bitte gegebenenfalls korrigieren.*

<Nachname>, <Vorname> ____________________

<Geburtsdatum> ____________________

<Straße>, <Hausnummer>, <Zusatz> ____________________

<PLZ>, <Wohnort> ____________________

Diagnose: <Diagnose_Patient> ____________________

**Dieser Bogen wird für jede Patientin/ jeden Patienten nur einmal erhoben.**

**Aktuell bekannte Komorbiditäten** (Mehrfachnennung möglich)**:**

☐ Arterielle Hypertonie
☐ Herzvitien/ Kardiomyopathie
☐ Koronare Herzkrankheit, Myokardinfarkt
☐ Herzinsuffizienz
☐ Schlaganfall, TIA u.ä.
☐ Periphere Gefäßerkrankungen
☐ Chronisch obstruktive Lungenerkrankung (COPD, Asthma bronchiale)
☐ Niereninsuffizienz
☐ Rheumatoide Arthritis oder andere Autoimmunerkrankungen
☐ Schilddrüsenerkrankungen
☐ Diabetes mellitus
☐ Erkrankungen der Leber
☐ Demenz
☐ Sonstiges: ________________________________________

________________________________________________

**Vorliegen weiterer bösartiger Tumorerkrankungen:**

☐ Nein ☐ Ja, ☐ unbekannt

und zwar:___________________________________

Jahr der Erstdiagnose (wenn bekannt): __________

**Bitte beantworten Sie jetzt Fragen zur Nachsorge bei Ihrer Patientin/ Ihrem Patienten.**

**Führen Sie die Nachsorgeuntersuchungen regelmäßig durch?**

☐ Nein ☐ Ja

**Wie häufig haben Sie Ihre Patientin/ Ihren Patienten in den letzten 12 Monaten zur Nachsorgeuntersuchung einbestellt?**

☐ Jährlich

☐ Halbjährlich

☐ Vierteljährlich

☐ Häufiger,
und zwar: ___________________

☐ Unregelmäßig

**Führen Sie im Rahmen der Nachsorge mit Ihrer Patientin/ Ihrem Patienten Gespräche über allgemeine Vorsorgemaßnahmen?**

**Über Krebsfrüherkennungsuntersuchungen?**

☐ Nie ☐ Gelegentlich ☐ Immer

**Über den Impfstatus?**

☐ Nie ☐ Gelegentlich ☐ Immer

**Über kardiovaskuläre Risikofaktoren?**

☐ Nie ☐ Gelegentlich ☐ Immer

**Über andere Vorsorgemaßnahmen?**

Welche?_________________________________________________________________

☐ Nie ☐ Gelegentlich ☐ Immer

**Bitte schätzen Sie die Bedeutung der einzelnen Aspekte der Nachsorge zum gegenwärtigen Zeitpunkt bei dieser Patientin/ diesem Patienten ein.**

| | | sehr wichtig | eher wichtig | eher unwichtig | völlig unwichtig |
|---|---|---|---|---|---|
| Erkennen von Rezidiven | | ☐ | ☐ | ☐ | ☐ |
| Erkennen kardiovaskulärer Folgeerkrankungen | | ☐ | ☐ | ☐ | ☐ |
| Erkennen von Sekundärmalignomen | | ☐ | ☐ | ☐ | ☐ |
| Erfassen von Fertilitätsproblemen | | ☐ | ☐ | ☐ | ☐ |
| Erfassen infektiöser Komplikationen | | ☐ | ☐ | ☐ | ☐ |
| Erfassen von Polyneuropathien | | ☐ | ☐ | ☐ | ☐ |
| Aspekte der allgemeinen Gesundheits-fürsorge | Planen empfohlener Krebsfrüherkennungs-untersuchungen | ☐ | ☐ | ☐ | ☐ |
| | Prüfen des Impfstatus | ☐ | ☐ | ☐ | ☐ |
| | Erfassen und ggf. Behandeln kardiovaskulärer Risikofaktoren | ☐ | ☐ | ☐ | ☐ |
| Erfassen psychosozialer Probleme | | ☐ | ☐ | ☐ | ☐ |
| Sonstiges: ______________________ ______________________ | | ☐ | ☐ | ☐ | ☐ |

**Datum des letzten Kontakts zu Ihrer Patientin/ Ihrem Patienten:** ______________________

**Heutiges Datum:** ______________________

**Nächster geplanter Nachsorgetermin:** ______________________

(Diese Angabe ist für uns wichtig, da wir uns zur Dokumentation der dann durchgeführten Maßnahmen erneut mit Ihnen in Verbindung setzen werden.)

**Herzlichen Dank für Ihre Mühe!**

Bitte im vorbereiteten Freiumschlag zurücksenden an das:

Zentrum für klinische Studien (ZKSE)

c/o Institut für medizinische Informatik, Biometrie und Epidemiologie (IMIBE)

Stichwort: ABC-Studie

Hufelandstraße 55

45147 Essen

Universitätsklinikum Essen

westdeutsches tumorzentrum

GEFÖRDERT VOM Bundesministerium für Bildung und Forschung

**ABC-Studie**

**Klinik für Hämatologie**
Dir.: Prof. Dr. med. U. Dührsen

**Nachsorgemuster bei erwachsenen Patientinnen und Patienten mit Blutkrebs und Blutkrebsvorstufen: Einfluss auf gesundheitsbezogene Endpunkte, Lebensqualität und Ressourcennutzung**

# Arztfragebogen zur Nachsorge
# Aktuelle Nachsorge

**Ihre Patientin/ Ihr Patient:**

*Sind die Angaben zu Ihrer Patientin/ Ihrem Patienten korrekt? Bitte gegebenenfalls korrigieren.*

<Nachname>, <Vorname> ____________________

<Geburtsdatum> ____________________

<Straße>, <Hausnummer>, <Zusatz> ____________________

<PLZ>, <Wohnort> ____________________

**Datum aktueller Nachsorgetermin:** ____________________

**Datum letzter Nachsorgetermin:** ____________________

**Der Inhalt dieses Fragebogenabschnitts bezieht sich auf die aktuelle Nachsorgeuntersuchung bzw. auf die Zeit seit der letzten Nachsorgeuntersuchung.**

**Durchgeführte Untersuchungen** (Mehrfachnennung möglich)**:**

☐ Ärztliches Gespräch
☐ Körperliche Untersuchung
☐ Blutentnahme
☐ Blutdruckmessung
☐ EKG
☐ Abdomensonographie
☐ Lymphknotensonographie
☐ Röntgenuntersuchung des Thorax
☐ Röntgenuntersuchung des Skelettsystems
☐ Echokardiographie
☐ Computertomographie von: ____________________
☐ Kernspintomographie von: ____________________
☐ Positronenemissionstomographie
☐ Knochenmarkpunktion

☐ Sonstiges: ____________________

____________________

**Aspekte, die im aktuellen ärztlichen Gespräch thematisiert wurden:**

| | | durch den Arzt/ die Ärztin | durch die Patientin/ den Patienten |
|---|---|---|---|
| Hinweise auf ein Rezidiv der Erkrankung (B-Symptomatik, Lymphknotenschwellungen, Blutungszeichen, usw.) | | ☐ | ☐ |
| Neu aufgetretene Begleiterkrankung (seit der letzten Nachsorgeuntersuchung) | | ☐ | ☐ |
| Hinweise auf eine neu aufgetretene kardiovaskuläre Erkrankung (Ruhe-/ Belastungsdyspnoe, Angina pectoris, Ödeme) | | ☐ | ☐ |
| Sekundärmalignome | | ☐ | ☐ |
| Probleme mit der Sexualität | | ☐ | ☐ |
| Infertilität | | ☐ | ☐ |
| Gesteigerte Infektanfälligkeit | | ☐ | ☐ |
| Polyneuropathie | | ☐ | ☐ |
| Aspekte der allgemeinen Gesundheits-fürsorge | Allgemeine Krebsvorsorge | ☐ | ☐ |
| | Impfungen | ☐ | ☐ |
| | Kardiovaskuläre Risikofaktoren | ☐ | ☐ |
| Psychosoziale Probleme | | ☐ | ☐ |
| Sonstiges: ____________________ ____________________ | | ☐ | ☐ |

**Angeordnete Laboruntersuchungen** (Mehrfachnennung möglich)**:**

☐ keine

☐ Blutbild/ Differentialblutbild
☐ Gerinnungsparameter
☐ Nierenretentionsparameter
☐ Leberwerte
☐ Elektrolyte
☐ LDH
☐ Gesamteiweiß
☐ Serumeiweißelektrophorese
☐ Immunglobuline quantitativ
☐ Freie Leichtketten
☐ Immunfixation

☐ Zelluläres Immunogramm
☐ Vitamin B12/ Folsäure
☐ Eisen/ Ferritin/ Transferrin
☐ Tumormarker (ß2-Mikroglobulin, Thymidinkinase, usw.)
☐ Hormone, welche: ____________________
☐ Urinstatus
☐ 24-Std-Sammelurin auf: ____________________

☐ Sonstige: ____________________________________________

____________________________________________

**Aktueller Tumorstatus:**

☐ Komplette Remission
☐ Partielle Remission
☐ Stabile Erkrankung
☐ **Nicht** therapiebedürftiges Rezidiv/ nicht therapiebedürftiger Progress
☐ Therapiebedürftiges Rezidiv/ therapiebedürftiger Progress

**Haben sich in der aktuellen Nachsorgeuntersuchung Hinweise auf therapie-/ krankheitsbedingte Komplikationen ergeben.**
**Wenn ja, schildern Sie bitte kurz die Symptome, die veranlasste Diagnostik und die evtl. eingeleitete Therapie.**

____________________________________________

____________________________________________

____________________________________________

____________________________________________

____________________________________________

**Gab es außerplanmäßige Vorstellungen der Patientin/ des Patienten seit dem vorherigen geplanten Nachsorgetermin?**

☐ Nein
☐ Ja, aufgrund von: __________________________
☐ ambulant
☐ stationär

**Im Rahmen der aktuellen Nachsorge wurden Gespräche zu folgenden allgemeinen Früherkennungs- und/oder Vorsorgemaßnahmen geführt:**

**Zu Krebsfrüherkennungsuntersuchungen?**

☐ Hämoccult-Test
☐ Koloskopie
☐ Hautkrebs-Screening
☐ Brustkrebs-Screening/ Mammographie-Screening
☐ Zervixkarzinom-Screening
☐ Prostatakarzinom-Screening mittels PSA-Bestimmung

☐ Sonstiges: ____________________________________

____________________________________

**Zum Impfstatus?**

☐ Tetanus
☐ Diphtherie
☐ Influenza
☐ Pneumokokken
☐ Hämophilus influenzae Typ B
☐ Meningokokken
☐ Hepatitis A
☐ Hepatitis B

☐ Sonstiges: ____________________________________

____________________________________

**Zu kardiovaskulären Risikofaktoren?**

☐ Adipositas ☐ Arterielle Hypertonie ☐ Diabetes mellitus
☐ Rauchen ☐ Hyperlipidämie

☐ Sonstiges: ________________________________________________

________________________________________________

**Seit dem letzten Nachsorgetermin wurden folgende Nachsorgemaßnahmen für die Blutkrebserkrankung oder für eine Folge der Blutkrebserkrankung verordnet** (Mehrfachnennung möglich)**:**

**Reha-Maßnahmen** (Dauer in Tagen)**:**

☐ Keine

☐ stationär Aufenthalt 1:________ Tage Aufenthalt 2:________ Tage

☐ ambulant Maßnahme 1:_______ Tage Maßnahme 2:_______ Tage

**Krankengymnastik oder ähnliche Heilverfahren**
(Einheiten entspricht Anzahl der Besuche)**:**

☐ Keine

☐ Physiotherapie ________ Einheiten

☐ Ergotherapie ________ Einheiten

☐ Logopädie ________ Einheiten

☐ Sonstiges ________ Einheiten

und zwar ________________________________________________

________________________________________________

**Seit dem letzten Nachsorgetermin wurden folgende Medikamente aufgrund der Blutkrebserkrankung oder einer Folge der Blutkrebserkrankung verordnet.**
(Bitte keine Begleitmedikation zu Komorbiditäten angeben.)**:**

☐ Keine

| Präparat/ Wirkstoff | Darreichungs-form | Dosis pro Einheit | Einnahme-zeitraum (Dauer in Tagen) | Tägliches Dosierungs-schema (z.B. 1-0-1) |
|---|---|---|---|---|
| | | | | |
| | | | | |
| | | | | |
| | | | | |
| | | | | |
| | | | | |
| | | | | |
| | | | | |
| | | | | |
| | | | | |
| | | | | |
| | | | | |
| | | | | |

**Heutiges Datum:** ________________________________

**Nächster geplanter Nachsorgetermin:** ________________________________

(Diese Angabe ist wichtig, da wir uns zur Dokumentation der dann durchgeführten Maßnahmen erneut mit Ihnen in Verbindung setzen werden.)

**Herzlichen Dank für Ihre Mühe!**

Bitte im vorbereiteten Freiumschlag zurücksenden an das:

Zentrum für klinische Studien (ZKSE)

c/o Institut für medizinische Informatik, Biometrie und Epidemiologie (IMIBE)

Stichwort: ABC-Studie

Hufelandstraße 55

45147 Essen
